# Prognostic relevance of PRSS2 and its immune correlates in papillary thyroid carcinoma

**DOI:** 10.1515/med-2025-1283

**Published:** 2025-10-23

**Authors:** Wei Lin, Linwen Zeng, Xiaoxiao Jiang, Xiangdong Kong, Jianming Gong, Ming Wu

**Affiliations:** Department of Science and Education, Tinglin Hospital of Jinshan District, Shanghai, China; Department of Surgery, Tinglin Hospital of Jinshan District, Shanghai, China; Department of Nursing, Tinglin Hospital of Jinshan District, Shanghai, China

**Keywords:** PRSS2, papillary thyroid carcinoma, prognostic value, *TRBV*, *TSC22D1*, *CD40*

## Abstract

**Background:**

Papillary thyroid carcinoma (PTC) generally exhibits favorable prognosis; however, a subset of patients remains at risk for recurrence. Serine protease 2 (PRSS2) was an oncogenic factor in several solid tumors, yet its expression profile and functional role in PTC remain poorly defined. This study aimed to investigate the expression level of PRSS2 in PTC and its prognostic significance, as well as explore its potential involvement in immune regulatory mechanisms.

**Methods:**

PTC specimens from thyroidectomy patients were analyzed by transcriptomic analysis, quantitative real-time PCR, and immunohistochemistry. Differential gene expression and survival analyses were performed by integrating data from TCGA and GEO databases. Pearson correlation analysis was utilized to evaluate associations between *PRSS2* and immune-related genes.

**Results:**

PRSS2 was upregulated in PTC tissues. High *PRSS2* expression was associated with better survival (HR = 3.253; 95% CI: 1.155–9.160), especially in patients aged ≥62 and stage II/III. Patients with low *PRSS2* and high *BRAF* expression exhibited a markedly reduced 5-year overall survival rate. *PRSS2* also showed significant positive correlations with multiple immune-related genes, including a moderate to strong correlation with T-cell receptor beta variable (*TRBV*) region genes (*R* = 0.58–0.72), *CD40*, and transforming growth factor beta-stimulated clone 22 domain 1.

**Conclusions:**

PRSS2 is upregulated in PTC and is associated with favorable prognosis. Its association with *TRBV* and other immune-related genes suggests a correlation with tumor immune microenvironment. Further studies are needed to elucidate the biological functions of PRSS2 in PTC and to assess therapeutic potential.

## 1 Introduction

Papillary thyroid carcinoma (PTC), while generally associated with a favorable prognosis, exhibits marked clinical heterogeneity. A subset of patients presents with highly aggressive clinical features, accounting for nearly half of PTC-related mortality  [1,2]. This underscores the importance of identifying molecular biomarkers with predictive and prognostic value, which could enable more precise risk stratification in clinical practice [3].

Serine protease 2 (PRSS2), also known as anionic trypsinogen or trypsinogen-2, is a zymogen that can be proteolytically activated into tumor-associated trypsin-2 (TAT-2), an enzymatically active trypsin isoform implicated in protein degradation. Originally identified in the pancreas, aberrant overexpression of PRSS2 has been shown to induce pancreatitis [4]. Subsequent studies have confirmed that PRSS2 is also expressed in the gastrointestinal tract, urine, serum, lung tissue, vitreous humor, retina, immune, and inflammatory cells. Clinically, PRSS2 is significantly upregulated in various digestive system tumors (e.g., pancreatic, gastric, cholangiocarcinoma, and colorectal cancers) [5–9], as well as in non-digestive system tumors such as breast, prostate, and ovarian cancers [10,11]. And, high *PRSS2* expression is strongly associated with poor prognosis in gastric [9], breast, and prostate cancers [11]. Moreover, elevated serum levels of TAT-2 have been identified as an adverse prognostic factor in colorectal cancer patients over the age of 66, particularly in cases involving left-sided lesions  [8].

According to the “seed and soil” hypothesis, tumor progression is determined by both the intrinsic properties of cancer cells (the “seed”) [12] and the surrounding tumor microenvironment (the “soil”)  [13]. Intriguingly, PRSS2 has also been implicated in immune rejection during organ transplantation [14] and in hyperactivation of CD8⁺ T cells [15], suggesting that its role in tumorigenesis may be more complex. Nevertheless, the clinical significance and regulatory mechanisms of PRSS2 expression in PTC remain poorly understood and require further investigation.

In this study, we compared the expression levels of PRSS2 between PTC tissues and adjacent non-tumor tissues and assessed its association with prognosis using data from the TCGA database. Notably, although PRSS2 expression was upregulated in PTC, it was associated with favorable clinical outcomes. Given the relatively indolent character of PTC, we further explored the potential functional role of *PRSS2* in PTC pathophysiology through data mining and literature analysis.

## 2 Materials and methods

### 2.1 Collection and preservation of samples

Patient specimens were collected following approval from the TingLin Hospital and after obtaining written informed consent, during the period from January 1, 2022 to December 31, 2023. Twenty-two cases of PTC were diagnosed by two independent pathologists using histopathology. None of the patients had received preoperative radiotherapy, immunotherapy, or neoadjuvant chemotherapy. All patients underwent thyroidectomy. Freshly resected specimens were promptly snap-frozen in liquid nitrogen for preservation.

### 2.2 RNA-seq analysis and differentially expressed gene (DEGs) identification

Tissue specimens were homogenized in liquid nitrogen, followed by total RNA extraction with TRIzol. RNA purity and integrity were subsequently evaluated using a NanoDrop spectrophotometer. mRNA was enriched using Oligo(dT) magnetic beads, followed by fragmentation with the NEBNext Magnesium RNA Fragmentation Module (New England Biolabs, E6150S). cDNA was generated from the fragmented RNA and amplified to construct sequencing libraries with an average insert size of approximately 300 ± 50 bp. Sequencing was performed on the Illumina NovaSeq™ 6000 platform (LC Bio Technology Co., Ltd, Hangzhou, China).

Raw reads were subjected to quality control to remove adapter sequences and low-quality reads, generating clean reads. These clean reads were aligned to the reference genome using Hisat2. Intergroup comparisons were performed using Student’s *t*-test, and genes with *P* < 0.05 were subjected to false discovery rate (FDR) correction using the Benjamini–Hochberg method. DEGs were defined as those with FDR <0.05 and |log₂(fold change)| > 1.0. Gene expression levels were quantified as fragments per kilobase per million mapped fragments (FPKM). Functional annotation of DEGs was performed using the Gene Ontology (GO) database, and signaling pathway enrichment analysis was conducted using the Kyoto Encyclopedia of Genes and Genomes (KEGG) database.

### 2.3 Quantitative reverse transcription-polymerase chain reaction (qRT-PCR)

Using the RNA-Quick Purification Kit (Yishan Biotechnology, Cat# ES-RN001) from Shanghai Yishan Biotechnology Co., Ltd, total RNA was extracted. The PrimeScript RT Master Mix from Roche Diagnostics in Basel, Switzerland, was used for reverse transcription. PCR was performed using BeyoFast SYBR Green qPCR Mix (2X; High ROX; Beyotime Biotechnology Inc., Shanghai, China). Sequences of PCR primers were CAGCCGGACTCTGGACAATG (forward) and GACACGCGGGAATTGATGAC (reverse) for *PRSS2* and TCATCACCATTGGCAATGAG (forward) and CACTGTGTTGGCGTACAGGT (reverse) for β-actin. After initial denaturation at 95°C for 1 min, the amplification was performed with 40 cycles of denaturation at 95°C for 10 s and annealing/extension at 60°C for 30 s. The threshold cycle (Ct) was determined using the 7300 real-time PCR system (version 1.4, Applied Biosystems; Thermo Fisher Scientific, Inc., Waltham, MA, USA). β-actin was used as an internal control for normalization and 2^−(∆∆Ct)^ was calculated for the expression level of target genes. Experiments were repeated at least three times.

### 2.4 Immunohistochemistry (IHC) staining

IHC was performed with slight modifications as previously described in the literature [16]. In brief, the primary antibody applied was anti-PRSS2 (Proteintech, 15005-1-AP), and the negative control was phosphate-buffered saline. PRSS2 positivity was classified as 0 (0–5%), 1 (5–25%), 2 (26–50%), 3 (51–75%), and 4 (76–100%) according to the percentage of tumor cells. The intensity of immunostaining was classified as 0 (negative), 1 (weak), 2 (moderate), and 3 (strong). The final PRSS2 expression was low (0–2) or high (3–7) according to the sum of the positive cell rate and staining intensity score.

### 2.5 DEG expression analysis

Gene expression level of *PRSS2* and B-Raf proto-oncogene (*BRAF*) were obtained from the TCGA database (https://portal.gdc.cancer.gov/). RNA-seq data, originally in FPKM format, were converted to transcripts per million (TPM) reads format and subsequently log2-transformed using the R software package. GraphPad Prism 8.0.2 was utilized for data visualization. Based on ascending TPM values, a series of cutoffs were applied to stratify patients into high- and low-expression groups, followed by 5-year overall survival analyses. The cutoff was iteratively adjusted, and *P*-values were calculated using the log-rank test. The cutoff yielding the minimum *P*-value corresponded to TPM = 1.1 for *PRSS2* and TPM = 8.0 for *BRAF*, which were defined as the optimal thresholds. About 59% of patients (*n* = 290) and 58% of patients (*n* = 289) were classified into the *PRSS2* and *BRAF* high-expression groups, respectively.

### 2.6 cBioPortal for cancer genomics dataset

cBioPortal (http://cbioportal.org) draws data from several authoritative databases, including the Gene Expression Omnibus (GEO; http://www.ncbi.nlm.nih.gov/geo/) and TCGA database. cBioPortal is a web resource for exploring, visualizing, and analyzing multidimensional cancer genomics data. The genomic profile of each gene includes mutations, putative copy-number alterations, and mRNA expression *z*-scores. The *z* score for each gene represents the normalized mRNA expression calculated using RNA-seq by expectation-maximization method.

### 2.7 Statistical analysis

All data were processed with the software GraphPad Prism 8.0.2. Student’s *t*-test and paired Student’s *t*-test were carried out for comparisons between two groups. Survival curve analysis was performed using the log-rank test. The optimal cutoff value was determined by testing different expression thresholds. To investigate the intrinsic mechanisms of *PRSS2* expression in PTC tissues, Pearson coefficients were calculated to assess correlation between genes. When the absolute value of the Pearson’s correlation coefficient was larger, the correlation was stronger. This study selected genes with an absolute value of the Pearson’s correlation coefficient >0.3. *P* < 0.05 was considered to indicate a statistically significant difference.

## 3 Results

### 3.1 High expression of PRSS2 in PTC

**Figure 1:**
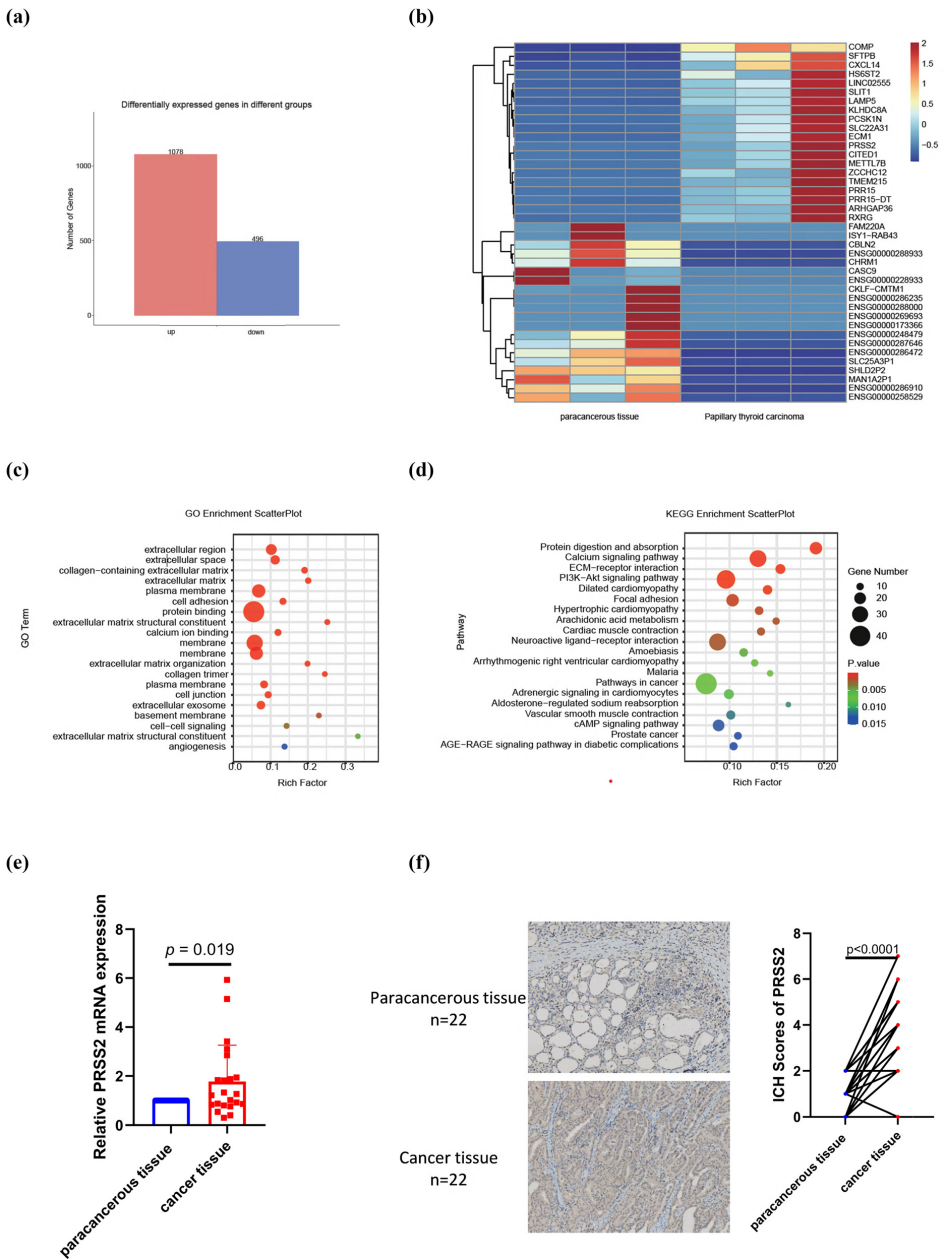
PRSS2 expression in papillary thyroid carcinoma. (a) Statistical analysis and (b) clustering heatmap of DEGs based on RNA-seq data comparing PTC tissues and adjacent non-tumorous tissues. (c) GO enrichment analysis and (d) KEGG pathway classification and functional enrichment of the DEGs. (e) qRT-PCR and (f) immunohistochemical analysis of PRSS2 expression in PTC and adjacent non-tumorous tissues.

The next-generation sequencing result showed that, a total of 1,574 DEGs were identified in PTC compared to paracancerous tissues, including 1,078 upregulated and 496 downregulated DEGs (Figure 1a and b). Subsequent enrichment analysis was performed on the DEGs. GO enrichment revealed that most terms were associated with the extracellular microenvironment including protein binding, extracellular region/space, extracellular matrix, and collagen-containing extracellular matrix (Figure 1c). KEGG pathway analysis demonstrated significant enrichment in pathways related to extracellular matrix remodeling and signal transduction, such as protein digestion and absorption, ECM–receptor interaction, and the PI3K–Akt signaling pathway (Figure 1d). The qRT-PCR (*n* = 22) and IHC (*n* = 22) results were consistent with the RNA-Seq data, indicating that PRSS2 expression was markedly elevated in PTC compared to paracancerous tissues (Figure 1e and f). Additionally, serum PRSS2 levels showed no significant difference between 23 PTC patients and 26 controls (data not shown).

### 3.2 High *PRSS2* expression is significantly associated with improved survival in PTC

**Figure 2:**
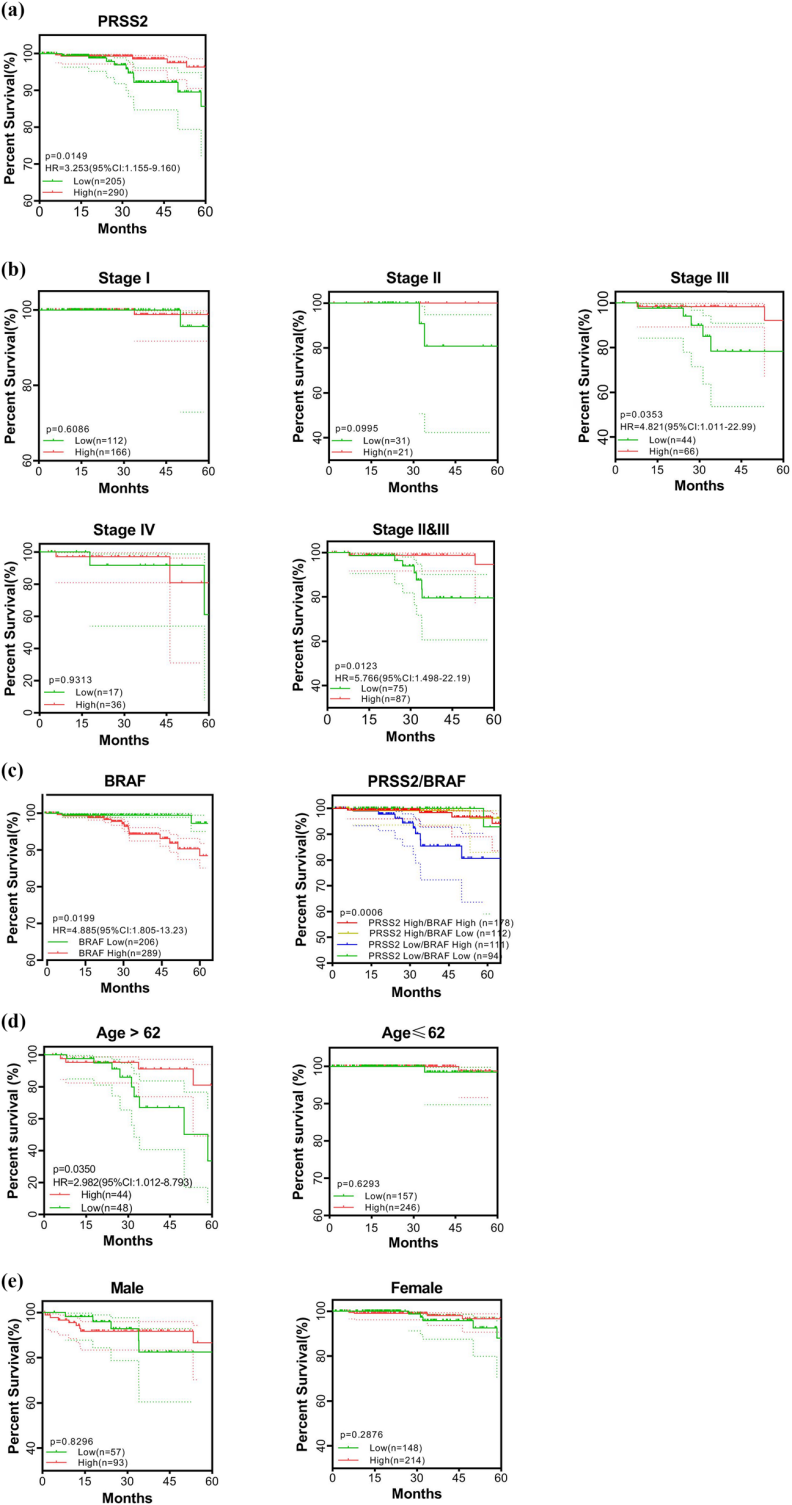
Prognostic significance of *PRSS2* in papillary thyroid carcinoma patients. (a) Overall survival analysis stratified by *PRSS2* expression levels. (b) Subgroup analyses of overall survival based on *PRSS2* expression and clinical stage. (c) Overall survival analysis stratified by *BRAF* expression levels (left) and subgroup analyses on *PRSS2/BRAF* expression (right). (d) and (e) Subgroup analyses of overall survival based on *PRSS2* expression, further stratified by age (d) and sex (e). HR: hazard ratio; CI: confidence interval.

Using tissue samples from 495 PTC patients in TCGA, patients were continuously stratified by *PRSS2* expression, identifying TPM = 1.1 as the optimal prognostic cutoff. Subsequently, clinical data from 290 patients with high *PRSS2* expression and 205 with low expression were analyzed to assess its prognostic relevance in PTC. High *PRSS2* expression was significantly associated with better survival percent (HR = 3.253; 95% CI: 1.155–9.160; *P* = 0.0149, Figure 2a).

Based on the optimal *PRSS2* stratification, multivariate analysis incorporating patient age, sex, and clinical stage was conducted to identify the most significant factor combinations influencing prognosis associated with *PRSS2* expression levels. In Figure 2b, analysis of overall survival stratified by *PRSS2* expression levels and clinical stage showed that in stage III patients, high *PRSS2* expression correlated with improved survival percent (*P* = 0.0353), whereas in stage II, a similar trend was observed but did not reach statistical significance (*P* = 0.0995, Figure 2b). No statistical association was found in stage I or IV (*P* = 0.6086 and 0.9313, respectively, Figure 2b). Clinically, stage I PTC is generally characterized by localized tumors without aggressive evidence, whereas stage II and III tumors may still be confined to the thyroid gland but exhibit more aggressive behavior. Stage IV typically indicates extrathyroidal extension or distant metastasis. Based on this clinical rationale, we combined stage II and III cases and observed a significant survival advantage in patients with high *PRSS2* expression (*P* = 0.0123, Figure 2b).

Analysis of *BRAF* expression revealed that high expression correlates with poorer prognosis (*P* = 0.0199, Figure 2c). Further stratification combining *PRSS2* and *BRAF* expression divided PTC patients into four subgroups, with the *PRSS2* low/*BRAF* high group exhibiting the lowest 5-year overall survival, while the other three groups showed similar outcomes (*P* = 0.0006, Figure 2c). This stratification demonstrates strong potential as a prognostic indicator.

In age-stratified analysis, high *PRSS2* expression was linked to better prognosis in patients over 62 years (HR = 2.982; 95% CI: 1.012–8.793; *P* = 0.0350, Figure 2d), but not in younger individuals (*P* = 0.6293, Figure 2d). Despite a higher incidence of PTC in females, *PRSS2* expression was not significantly associated with survival in either sex (Figure 2e).

### 3.3 DEGs in PTC and GO and KEGG enrichment analyses

**Figure 3:**
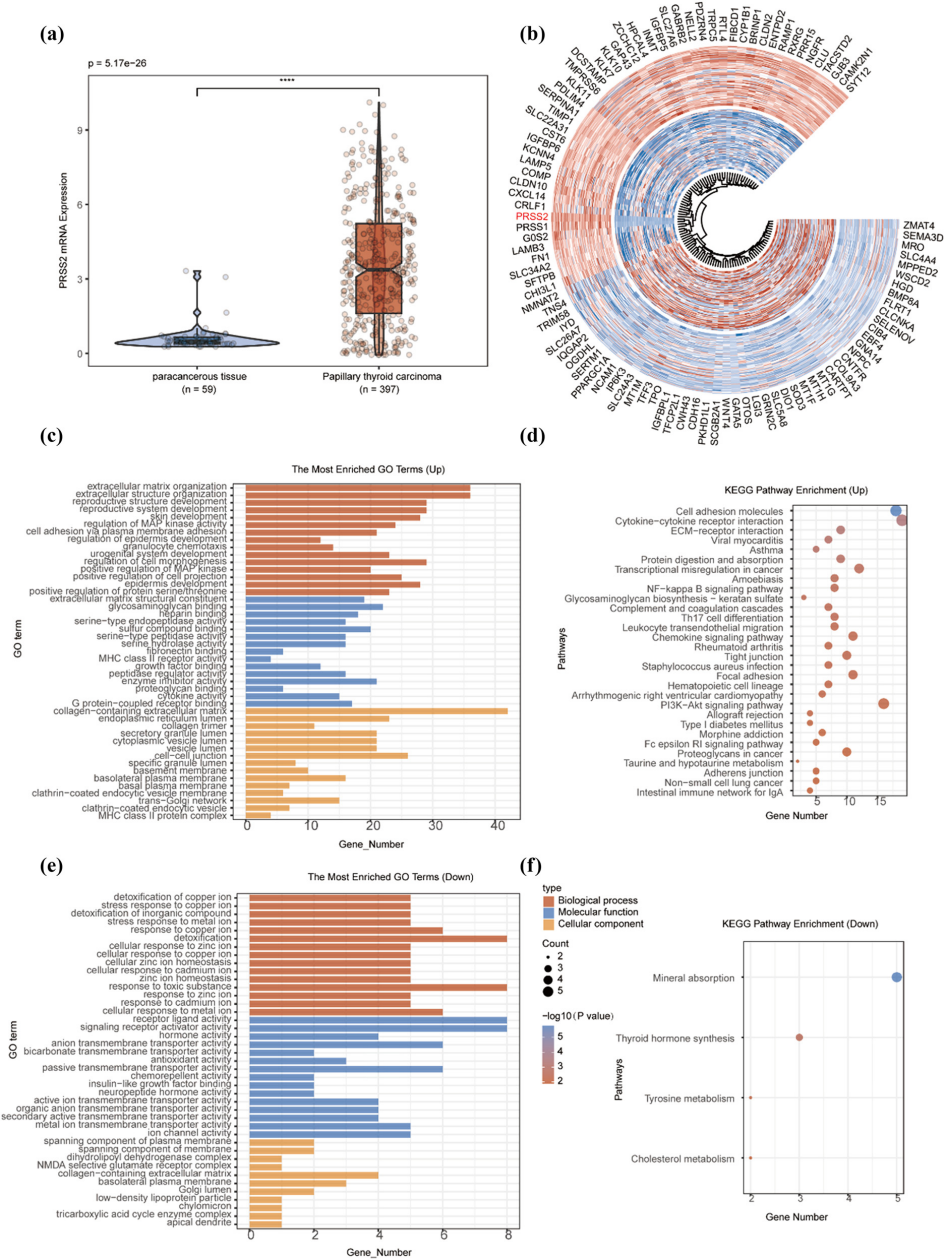
DEGs analysis of papillary thyroid carcinoma based on TCGA database. (a) Statistical analysis of DEGs between papillary thyroid carcinoma and adjacent normal tissues. (b) Heatmap of the top 50 significantly upregulated and downregulated DEGs. (c)–(f) Functional enrichment analysis of upregulated and downregulated genes based on KEGG pathway and GO term annotations.

DEGs analysis between PTC and adjacent non-tumorous tissues was conducted using data from both the TCGA (Figure 3a and b) and GEO (Figure S1a and b) databases. The results revealed that *PRSS2* was significantly upregulated in PTC (Figure 3b). GO and KEGG enrichment analyses were then performed on the DEGs, with results presented as dot plots (Figures 3c–f and S1c–f). Notably, among the top 30 KEGG pathways enriched by the upregulated DEGs in the TCGA dataset, 15 pathways (50%) were related to immune responses (Figure 3d). Similarly, in the GEO dataset, 12 out of top 20 pathways (60%) were immune-associated (Figure S1d). These indicated a pronounced immunological enrichment tendency. Several pathways, including complement and coagulation cascades, Th17 cell differentiation, leukocyte transendothelial migration, and hematopoietic cell lineage, were consistently enriched across both datasets.

### 3.4 *PRSS2* co-expression genes

**Figure 4:**
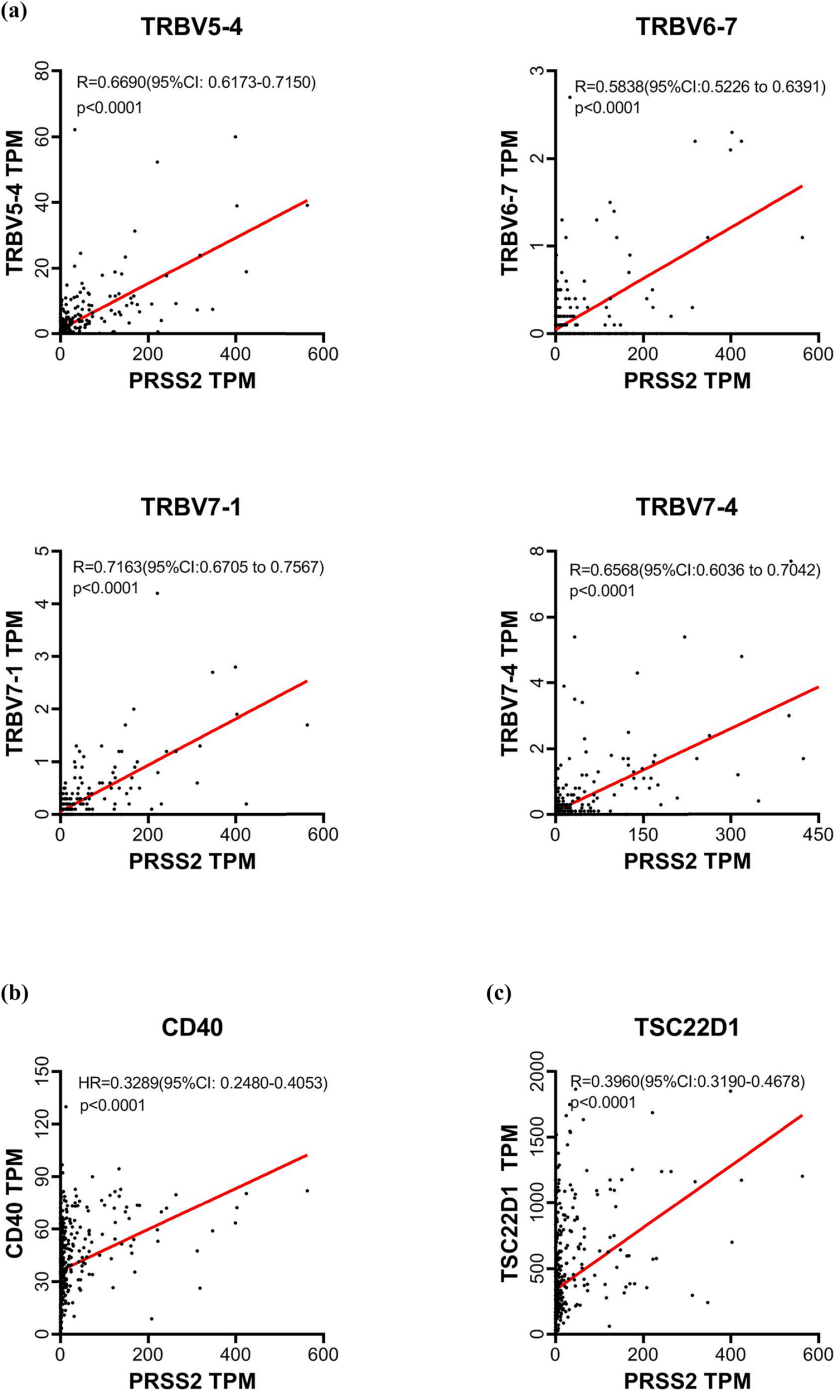
Correlation between *PRSS2* expression and immune activation-related genes. Scatter plots showing significant positive correlations between *PRSS2* expression and *TRBV* (a), *CD40* (b), and *TSC22D1* (c).

Given the involvement of PTC DEGs in immune-related regulation, we further analyzed the correlation between *PRSS2* and immune-associated genes using TCGA data and Pearson analysis. *PRSS2* expression was significantly positively correlated with several immune-related genes (*P* < 0.05), including T-cell receptor beta variable (*TRBV*) region genes (Figure 4a), *CD40* (Figure 4b), and transforming growth factor beta-stimulated clone 22 domain 1 (*TSC22D1*) (Figure 4c). Among them, *PRSS2* showed moderate to strong correlations with multiple *TRBV* genes (*R* = 0.58–0.72), with *TRBV7-1* exhibiting the highest correlation (*R* = 0.7163).

## 4 Discussion

Transcriptomic analysis revealed that PRSS2 expression is significantly elevated in PTC tissues compared to adjacent normal tissues, validated at both mRNA and protein levels. Analysis of TCGA database further indicated that high *PRSS2* expression correlates with better survival, particularly among elderly patients (≥62 years) and those at intermediate clinical stages (stage II and III).

However, previous studies have reported that elevated PRSS2 expression correlates with poor prognosis in gastric [9], breast, and prostate cancers [11], and promotes epithelial–mesenchymal transition in gastric cancer by upregulating MMP-9 [17]. In contrast, the association between high *PRSS2* expression and favorable prognosis in PTC suggests that its oncogenic role may not be straightforwardly evident in the context of PTC, and that its biological function might be influenced by the specific tumor microenvironment and pathological state.

Enrichment analysis of upregulated DEGs from GEO and TCGA datasets consistently highlighted strong immune-related signatures. Among the KEGG pathways significantly enriched in both databases were hematopoietic cell lineage, neutrophil (granulocyte chemotaxis, neutrophil degranulation, neutrophil activation involved in immune response), Th17 cell differentiation, leukocyte transendothelial migration, and complement and coagulation cascades – all of which represent classical immune regulatory mechanisms. Pathways enriched exclusively in a single database also demonstrated distinct immune relevance. In innate immunity, enriched pathways included phagosome, platelet degranulation, and virus receptor activity. For adaptive immunity, enrichment was observed in MHC class II receptor activity, immunoglobulin binding, Fc epsilon RI signaling pathway, intestinal immune network for IgA production, and allograft rejection. Additionally, pathways involved in immune cell recruitment and activation, such as cytokine–cytokine receptor interaction, chemokine signaling pathway, and cell adhesion molecules, were also significantly enriched. Inflammatory-related signaling cascades, including the NF-κB signaling pathway, asthma, and rheumatoid arthritis, as well as infection-related pathways tied to immune responses – tuberculosis, human papillomavirus infection, and pathogenic *Escherichia coli* infection – were likewise prominently enriched.

This widespread enrichment of immune-related pathways indicates that the immune microenvironment in PTC is not suppressed; on the contrary, it may be actively engaged. Such an immunological landscape could offer a plausible explanation for the observation that high *PRSS2* expression is associated with better prognosis in PTC. Indeed, PTC has been categorized as an “inflammatory” immune subtype, characterized by substantial immune cell infiltration within the tumor tissue [18], a notion supported by our findings. Furthermore, previous studies consistent with our view have reported that PTC tumors in patients <45 years old and in stage I/II harbor greater lymphocytic infiltration than those in older patients (≥45 years) or in advanced stages (III/IV) [19].

PRSS2 is linked to several enriched pathways mentioned above. PRSS2 is the most highly expressed serine protease in the hematopoietic microenvironment and closely related to hematopoietic lineages. PRSS2 shows exceptionally high expression in CD34⁺ hematopoietic stem cells, multipotent progenitors, and common myeloid progenitors, with levels 147-fold higher than in CD34⁻ cells and significantly exceeding other serine protease isoforms [20]. Moreover, PRSS2 is involved in allograft rejection; serum PRSS2 markedly increases during chronic antibody-mediated rejection in kidney transplant recipients [21]. These findings may provide insights into the concurrent immune pathway enrichment and PRSS2 overexpression in PTC.

Within this immunological context, *PRSS2* expression showed a strong positive correlation with several immune activation-related genes, including *TRBV*, *CD40*, and *TSC22D1*. Notably, the correlation with *TRBV* was the most pronounced. Pearson correlation analysis revealed moderate to strong positive associations between *PRSS2* and various *TRBV* gene segments, including *TRBV5-4*, *TRBV6-7*, *TRBV7-1*, and *TRBV7-4*, with *R*-value exceeding 0.58. In particular, the correlation between *PRSS2* and *TRBV7-1* reached *r* = 0.72, indicating a strong positive correlation.

The *TRBV* gene family encodes the variable regions of the T-cell receptor (TCR) β chain and is a key genetic module responsible for shaping TCR diversity and mediating antigen recognition by T lymphocytes. *TRBV* usage is tightly linked to immunological activity [22]. In anti-tumor immunity, T cells serve as the principal effector population of the adaptive immune response, and the functional engagement of TCRs – composed in part by *TRBV* segments – is essential for tumor antigen recognition and cytotoxic targeting [23–25]. Previous studies have demonstrated that restricted *TRBV* usage is associated with impaired T cell immunity [23] and correlates with reduced survival in tumor contexts [24].

It is noteworthy that both *PRSS2* and *TRBV* genes are located within chromosome7q35. In other species, such as rabbit (*Oryctolagus cuniculus*) and chicken (*Gallus gallus*), experimental evidence has demonstrated genomic colocalization of *PRSS2* with *TRBV* genes. For instance, in the *TCRβ* locus of chicken, three *PRSS2* genes are dispersed throughout the region, with one *PRSS2* gene situated immediately downstream of the *TRBV* gene, forming a repeated gene structure [26]. Similarly, in rabbits, five *PRSS* family genes have been identified within the *TCRβ* locus, with four residing downstream of the *TRBV1* gene [27]. In the human genome, *PRSS2* also resides downstream of the *TRBV* cluster. Moreover, the *PRSS1–PRSS2* haplotype overlaps extensive regions of the *TCRβ* locus [27].

Such colocalization implies possible regulatory interactions between *PRSS2* and *TRBV*, possibly influencing gene stability and transcriptional coordination. For example, in Philadelphia chromosome-negative adult T-cell acute lymphoblastic leukemia, biallelic deletion of *PRSS1* and *PRSS2* has been observed in patients with clonal rearrangements within the *TCRβ* locus [25]. Additionally, in pancreatitis, the *PRSS1–PRSS2* haplotype may affect *TRBV* composition and immunophenotype. Specifically, the expression of *TRBV29-1* has been reported to correlate more strongly with the *PRSS1–PRSS2* haplotype than with *PRSS2* expression itself  [28]. Collectively, the genomic colocalization of *PRSS2* and *TRBV* gene segments may partially explain the moderate-to-strong positive correlations observed in PTC, and further suggest a potential link between *PRSS2* expression and immune phenotype.

CD40 is a cell surface co-stimulatory receptor belonging to the tumor necrosis factor receptor superfamily. It is predominantly expressed on antigen-presenting cells, including dendritic cells, macrophages, and B lymphocytes, where it plays a pivotal role in modulating and activating both innate and adaptive immune responses [29]. CD40 has emerged as a promising target in tumor immunotherapy and has advanced to Phase I and II clinical trials. CD40 agonists have demonstrated antitumor efficacy in monotherapy (Phase I) [30] and enhanced immune responses in combination therapies (Phase II) [31]. In this study, *PRSS2* expression showed a positive correlation with *CD40*. Given the well-established role of CD40 in antitumor immunity, this correlation suggests that *PRSS2* may participate in the regulation of the tumor immune microenvironment. Supporting this, a study on canine insulinoma metastases reported a 20–70-fold upregulation of *PRSS2* transcripts, accompanied by localized expression of CD40–CD40L in the tumor microenvironment  [32]. Together, these findings provide preliminary evidence implicating PRSS2 in tumor–immune interactions.

*TSC22D1* is a member of the *TSC22D* family, is downregulated in glioblastoma, salivary gland, prostate, and cervical cancers [33]. It has been characterized as a candidate tumor suppressor and a transcriptional regulator [34]. The *TSC22D* family is known to contribute to cellular homeostasis and immune regulation [35]. Notably, *TSC22D3* (also known as *GILZ*) is a key mediator of glucocorticoid-induced immunomodulation and anti-inflammatory responses, and its expression is associated with immune cell infiltration  [36]. Moreover, in HIV-infected patients, the methylation status of *TSC22D1* has been linked to HIV-associated inflammation [37].

In contrast to gastric cancer, where *PRSS2* promotes tumor invasiveness activity, high *PRSS2* expression in PTC aligns with enhanced antitumor immunity activity. Tumor progression is driven by both intrinsic malignant traits and extrinsic microenvironmental factors. In PTC, the tumor microenvironment exhibits a relatively active immune profile. The significant correlations between *PRSS2* expression and a range of immune effector molecules suggest its potential involvement in immunoregulatory processes. Collectively, despite its high expression in PTC, *PRSS2* appears to be intricately involved in immune modulation, which may contribute to a more favorable tumor immune contexture and better clinical outcomes.

A limitation of this study is that no statistically significant difference in serum PRSS2 levels was observed between PTC patients and healthy controls within the small cohort. This may be due to PRSS2 expression across multiple normal tissues, with extremely high specificity in the pancreas, which heavily influences serum PRSS2 abundance. Since serum protein levels reflect cumulative expression from various tissues, the modest upregulation of PRSS2 in thyroid cancer tissue (approximately 2–4-fold compared to paracancerous tissue, Figure 1e and f) may be insufficient to notably alter serum concentrations and can be masked by high-expression tissues. Additionally, the small sample size may limit the detection of differences.

Furthermore, this retrospective study utilized tissue samples from surgically treated PTC patients, potentially introducing selection bias and limiting generalizability to the broader PTC population. Therefore, larger prospective, multicenter cohort studies combined with immune-related functional validation experiments are warranted to confirm these findings and elucidate underlying biological mechanisms.

## 5 Conclusion

This study demonstrates that PRSS2 is upregulated in PTC and is associated with improved prognosis in patients aged ≥62 years and those with stage II/III. Low *PRSS2*/high *BRAF* expression showed reduced 5-year overall survival. Its correlations with key immune-related genes, including *TRBV*, *CD40*, and *TSC22D1*, suggest that PRSS2 may be involved in tumor immune regulation, potentially contributing to improved clinical outcomes.

## Supplementary Material

Supplementary Figure
