# Supplementary material for: Prognostic relevance of PRSS2 and its immune correlates in papillary thyroid carcinoma
Source: Open Med (Wars). 2025 Oct 23;20(1):20251283. doi: 10.1515/med-2025-1283 (PMC12552859; doi:10.1515/med-2025-1283)
Supplement: Supplementary Figure [file med-2025-1283-sm.pdf]

Supplementary material

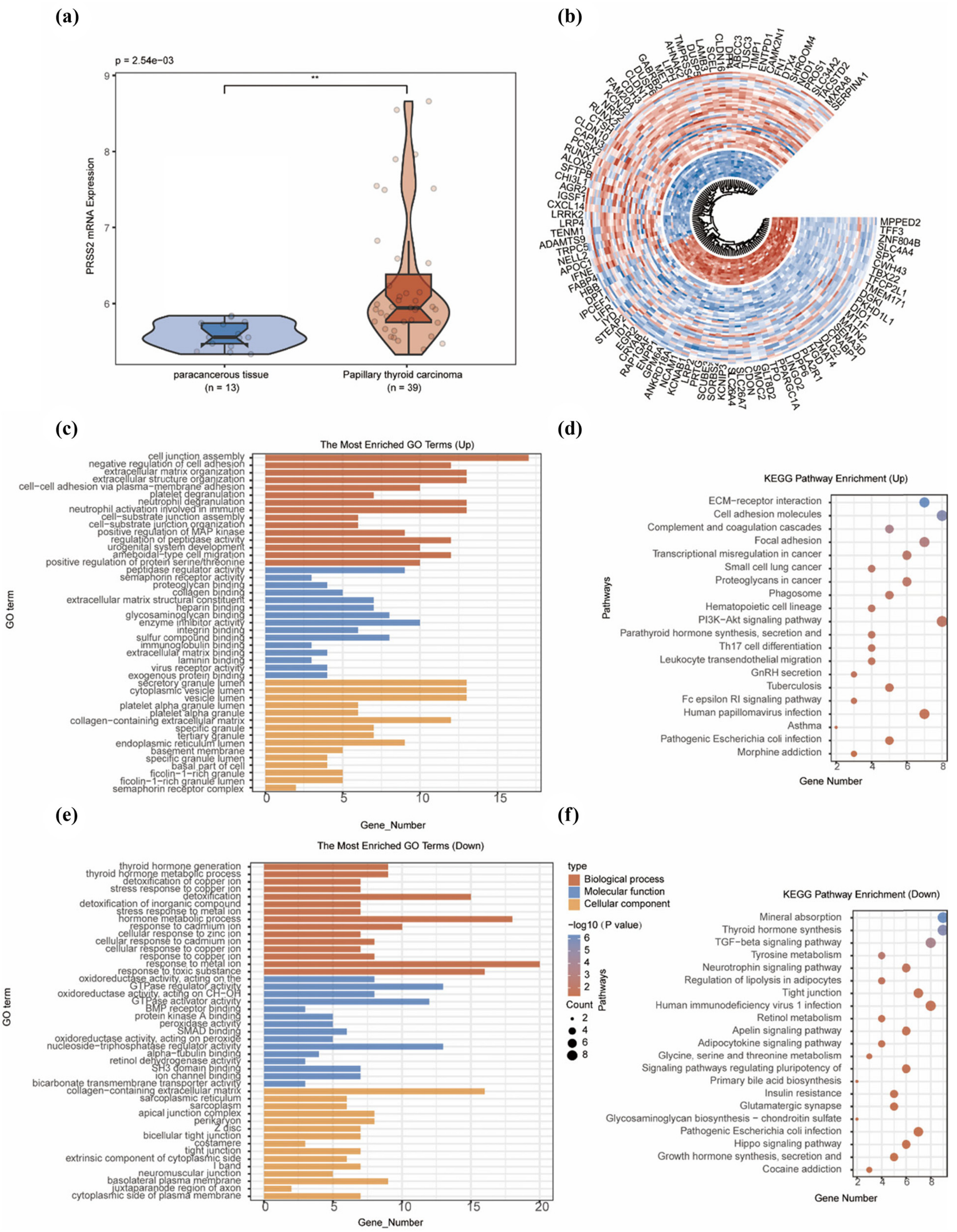

Figure S1: Differentially expressed genes (DEGs) analysis of papillary thyroid carcinoma based on GEO database. (a) Statistical analysis of DEGs between papillary thyroid carcinoma and adjacent normal tissues. (b) Heatmap of the top 50 significantly upregulated and downregulated DEGs. (c)–(f) Functional enrichment analysis of upregulated and downregulated genes based on KEGG pathway and GO term annotations.
